# Postural Stability, Rather than Strength–Coordination, Is Associated with Executive Functions in Preschool Children: A Structural Equation Modeling Study

**DOI:** 10.3390/children13070898

**Published:** 2026-07-06

**Authors:** Andrés Godoy-Cumillaf, Josivaldo de Souza-Lima, Frano Giakoni-Ramírez, Catalina Muñoz-Strale, Maribel Parra-Saldias, Daniel Duclos-Bastias, Claudio Farias-Valenzuela, Eugenio Merellano-Navarro, José Bruneau-Chávez

**Affiliations:** 1 Grupo de Investigación en Educación Física, Salud y Calidad de Vida (EFISAL), Facultad de Educación, Universidad Autónoma de Chile, Temuco 4780000, Chile; andres.godoy@uautonoma.cl; 2 Facultad de Educación y Ciencias Sociales, Instituto del Deporte y Bienestar, Universidad Andres Bello, Las Condes, Santiago 7550000, Chile; josivaldo.desouza@unab.cl (J.d.S.-L.); frano.giakoni@unab.cl (F.G.-R.); catalina.munoz@unab.cl (C.M.-S.); 3 Departamento de Educación Física, Deporte y Recreación, Universidad de Atacama, Copiapó 1530000, Chile; maribel.parra@uda.cl; 4 iGEO, Escuela de Educación Física, Facultad de Filosofía y Educación, Pontificia Universidad Católica de Valparaíso, Valparaíso 2340025, Chile; daniel.duclos@puvc.cl; 5 METIS Research Lab, Facultad de Negocios y Tecnología, Universidad Alfonso X el Sabio (UAX), 28691 Madrid, Spain; 6 Escuela de Ciencias de la Actividad Física, el Deporte y la Salud, Universidad de Santiago de Chile (USACH), Santiago 9170022, Chile; claudio.farias.v@usach.cl; 7 Department of Physical Activity Sciences, Faculty of Education Sciences, Universidad Católica del Maule, Talca 3530000, Chile; 8 Departamento de Educación Física, Deportes y Recreación, Universidad de la Frontera, Temuco 4811230, Chile; jose.bruneau@ufrontera.cl

**Keywords:** preschoolers, executive functioning, motor competence, balance control, structural equation modeling

## Abstract

**Highlights:**

**What are the main findings?**

- Postural stability, but not broader strength–coordination performance, was significantly associated with executive functions in preschool children.
- Physical activity and waist circumference were associated with executive functions primarily through their relationships with postural stability.

**What are the implications of the main findings?**

- Balance-related motor processes may represent an important domain for understanding motor–executive function associations during early childhood.
- Future longitudinal and experimental studies should further examine the role of postural stability in executive-function development during the preschool years.

**Abstract:**

Background/Objectives: Executive functions and motor performance develop rapidly during early childhood and may be closely interconnected. However, it remains unclear whether specific motor domains are more strongly associated with executive functioning than others. This study examined the relationships among executive functions, motor performance, physical activity, and waist circumference in preschool children using structural equation modeling (SEM). Methods: A cross-sectional study was conducted in 364 preschool children aged 4–7 years from Temuco, Chile. Executive functions were assessed using the Childhood Executive Functioning Inventory (CHEXI). Motor performance included postural stability and strength–coordination indicators derived from the PREFIT battery and the Movement Assessment Battery for Children-2. Physical activity was assessed using the Krece Plus questionnaire. SEM was used to examine direct and indirect statistical associations among variables while adjusting for age and sex. Results: The final SEM showed acceptable fit to the data (CFI = 0.915; TLI = 0.882; RMSEA = 0.054). Postural stability was significantly associated with executive functions (β = −0.268, *p* = 0.016), whereas strength–coordination was not. Physical activity positively predicted postural stability (β = 0.099, *p* = 0.024), while waist circumference negatively predicted postural stability (β = −0.095, *p* = 0.032). An indirect statistical association between waist circumference and executive functions through postural stability was observed. The model explained 9.6% of the variance in executive functions. Conclusions: In preschool children, executive functioning appeared to be more closely associated with postural stability than with broader strength–coordination performance. Although the observed associations were modest in magnitude, the findings suggest that balance-related motor processes may be relevant for understanding motor–executive function associations during early childhood. Longitudinal and experimental studies are needed to clarify the nature and direction of these associations.

## 1. Introduction

Executive functions are higher-order self-regulatory processes that support goal-directed behavior, particularly working memory, inhibitory control, and cognitive flexibility. These functions develop rapidly during the preschool years and are strongly associated with school readiness, learning, and later developmental outcomes [1,2]. Because early childhood represents a sensitive developmental window, identifying modifiable factors associated with executive functions during this stage is an important priority for pediatric and educational research [1,2].

A growing body of evidence suggests that executive functions develop in close interaction with motor behavior rather than in isolation. Recent systematic reviews and meta-analyses indicate that motor competence is positively associated with executive functions in children and adolescents [3], while preschool studies suggest that better motor competence and more physically active lifestyles are associated with better working memory and inhibitory control [4]. Experimental evidence further indicates that physical activity and exercise may benefit executive processes in children, although the magnitude of these associations varies according to age and intervention characteristics [5,6,7,8].

However, motor competence is not a unitary construct. It includes multiple dimensions such as balance, coordination, object control, and muscular performance, which may not be equally associated with executive functioning [3,4]. Postural stability may be especially relevant because balance control requires continuous sensory integration, anticipatory regulation, and inhibition of ineffective responses, processes conceptually related to executive regulation [9]. Emerging neurodevelopmental evidence provides additional support for this perspective. Postural control and executive functioning appear to rely, at least in part, on overlapping neural networks involving the cerebellum, prefrontal cortex, and their reciprocal connections [10,11]. Although traditionally viewed as a structure primarily involved in motor coordination, the cerebellum is increasingly recognized as contributing to higher-order cognitive processes through its interactions with frontal cortical regions [10,11]. Similarly, the prefrontal cortex plays a central role in executive control while also contributing to the regulation and adaptation of goal-directed motor behavior [10].

From an embodied cognition perspective, cognitive development is thought to emerge through continuous interactions between perception, action, and environmental demands [12]. Accordingly, motor experiences that require ongoing postural regulation, sensory integration, and adaptive responses may be particularly relevant for the development and expression of executive functions during early childhood [12]. By contrast, broader strength- or object-control performance may reflect more general physical abilities with less direct involvement of moment-to-moment self-regulation. Despite this rationale, relatively few studies in preschool children have examined whether postural stability and strength–coordination show distinct associations with executive functions within the same structural model [4].

Lifestyle and anthropometric factors may also contribute to these associations. Physical activity has consistently been associated with better executive functioning in childhood [5,6,13], whereas adiposity and waist circumference have been associated with poorer executive functioning and self-regulation [14,15]. Nevertheless, the mechanisms underlying these associations remain unclear, particularly during early childhood. One possibility is that motor functioning, especially balance-related performance, may be more closely associated with executive functioning than broader motor domains. However, although previous studies have reported associations between motor competence and executive functions [3,4,15], relatively few have simultaneously examined distinct motor domains, physical activity, anthropometric indicators, and executive functioning within a latent-variable framework.

Structural equation modeling provides an appropriate framework for addressing these questions because it allows the simultaneous estimation of measurement and structural relations while accounting for measurement error [16]. In addition, ecologically oriented tools such as the Childhood Executive Functioning Inventory (CHEXI) may be particularly useful in preschool populations because they assess executive-function difficulties as expressed in everyday contexts and have demonstrated adequate psychometric properties in young children [17].

Accordingly, the aim of the present study was to examine, in preschool children, the relationships among executive functions, motor performance, physical activity, and waist circumference using structural equation modeling. More specifically, we tested a model in which executive functions were represented by working memory and inhibition, motor performance was separated into postural stability and strength–coordination latent domains, and physical activity and waist circumference were evaluated as predictors of executive functions both directly and indirectly through motor factors. Based on theory and prior evidence, we hypothesized that postural stability, rather than strength–coordination, would show the strongest association with executive functions, and that physical activity and waist circumference would be more closely associated with executive functions through postural stability.

## 2. Materials and Methods

### 2.1. Study Design and Ethics

This study followed a cross-sectional observational design conducted in preschool children from Temuco, Chile, between September and December 2025. The study was approved by the Ethics Committee of Universidad Autónoma de Chile (protocol code N° 22–25; approved on 18 June 2025) and was conducted in accordance with the Declaration of Helsinki. Written informed consent was obtained from parents or legal guardians, and verbal assent was obtained from the children before participation.

### 2.2. Participants

A convenience sample of 364 preschool children aged 4–7 years was recruited from 8 preschool educational centers located in Temuco, Chile. The participating centers were situated in urban settings and included subsidized institutions serving children from predominantly middle socioeconomic backgrounds.

Recruitment was conducted through direct collaboration with the participating preschool centers. Parents or legal guardians received detailed information about the study objectives and procedures and were invited to allow their children to participate voluntarily. Children were eligible if they were between 4 and 7 years of age, attended one of the participating centers, and provided parental informed consent. Exclusion criteria included known neurological, developmental, musculoskeletal, or sensory conditions that could interfere with motor or cognitive assessments, acute illness on the day of evaluation, or missing data on the core study variables.

A total 450 children were initially invited to participate, of whom 402 agreed and completed the assessments, corresponding to a participation rate of approximately 89.3%. The final analytic sample included 364 children, of whom 53.0% were boys, with a mean age of 5.47 years (SD = 0.93).

### 2.3. Measures

#### 2.3.1. Executive Functions

Executive functions were assessed using the Childhood Executive Functioning Inventory (CHEXI), a parent-report questionnaire designed to evaluate executive-function difficulties in everyday contexts in children [17]. The CHEXI has been used in preschool populations and has demonstrated adequate psychometric properties, including acceptable reliability and factorial validity in young children [1,17].

The questionnaire was completed by parents or primary caregivers and evaluates behavioral manifestations of executive functioning observed in daily life situations. In line with previous validation studies, the present study focused on the two core executive-function domains most consistently identified in CHEXI research: working memory and inhibition [17].

Higher CHEXI scores indicate greater executive-function difficulties. Therefore, negative associations between motor-performance variables and CHEXI scores reflect better executive functioning. Working memory and inhibition scores were used as indicators of the latent Executive Functions construct in the SEM analyses.

#### 2.3.2. Motor Performance

Motor performance was assessed using field-based tests derived from the PREFIT battery and selected gross motor competence tasks from the Movement Assessment Battery for Children, second edition (MABC-2) [18,19]. The PREFIT battery has demonstrated adequate feasibility and reliability in preschool children [20,21].

Postural stability was assessed using static single-leg balance tasks performed separately on the right and left legs following standardized MABC-2 procedures [22]. Children were instructed to maintain balance on one leg for as long as possible, and performance time for each leg was used as an indicator of the latent factor Postural Stability.

The latent factor Strength–Coordination was represented by standing long jump, right- and left-hand grip strength, sprint performance, catching, and throwing tasks. Lower-body muscular strength was assessed using the standing long jump test from the PREFIT battery [18]. Upper-limb strength was evaluated using manual handgrip dynamometry (Takei 5401, Takei Scientific Instruments Co., Niigata, Japan). Grip strength was measured separately for the right and left hands following standardized procedures, and higher values indicated greater muscular strength.

Speed/agility was assessed using the 4 × 10 m sprint test from the PREFIT battery [18]. Sprint performance was reverse-coded before analysis so that higher values consistently reflected better motor performance across all indicators included in the SEM.

Gross motor object-control skills were assessed using the catching and aiming tasks from the MABC-2 [22], using the validated Spanish version of the instrument [23], which has demonstrated adequate psychometric properties (Cronbach’s α > 0.60; κ = 1; ICC = 0.85–0.99) [23]. Higher scores in the motor tasks reflected better motor performance.

To minimize measurement error, all assessments followed standardized administration procedures. Children wore comfortable sports clothing and appropriate footwear, and evaluators provided continuous encouragement and motivation throughout testing in accordance with PREFIT recommendations for preschool populations [18].

#### 2.3.3. Anthropometric Variables

Body height was measured using a stadiometer (Seca Model 200, seca GmbH & Co. KG, Hamburg, Germany ), and body weight was assessed using a digital scale (Tanita MC-780U, Tanita Corporation, Tokyo, Japan). Both measurements were obtained in duplicate, and the mean values were used to calculate body mass index (BMI) as weight divided by height squared (kg/m^2^).

Waist circumference was measured using a Rosscraft tape measure following standardized anthropometric procedures. The midpoint between the lower rib margin and the iliac crest was identified, and the measurement was obtained at the end of a normal expiration. Waist circumference was assessed three times, and the average value was used for analysis.

#### 2.3.4. Lifestyle Variables

Lifestyle indicators included parent-reported physical activity, screen time, sleep duration, and healthy eating score. Physical activity and lifestyle habits were assessed using the Krece Plus questionnaire, a brief instrument designed to evaluate lifestyle-related behaviors in pediatric populations [24]. The questionnaire includes items related to physical activity participation, sedentary behaviors, and dietary habits, with higher scores reflecting healthier lifestyle patterns.

In the final structural model, physical activity was retained as the main behavioral predictor because it showed the most consistent associations with the motor latent variables in preliminary analyses.

### 2.4. Model Specification

The analytical model was specified a priori to examine the relationships among executive functions, motor performance, physical activity, and anthropometric status in preschool children. Executive Functions were modeled as a latent construct indicated by working memory and inhibition, based on the two core domains captured by the Childhood Executive Functioning Inventory (CHEXI).

For the motor domain, two alternative conceptualizations were considered during model development. A unidimensional specification would have treated all motor indicators as reflecting a single general motor competence construct. However, based on theoretical considerations, the final model distinguished between two related but separable motor latent factors: Postural Stability, indicated by right- and left-leg balance performance, and Strength–Coordination, indicated by standing long jump, right- and left-hand grip strength, inverted sprint performance, catching, and throwing. This distinction was considered conceptually appropriate because balance tasks primarily reflect postural control, sensorimotor integration, and online regulatory adjustment, whereas the remaining tasks reflect broader neuromuscular and object-control performance.

Executive Functions and Postural Stability were each represented by two-indicator latent variables. Although latent factors are often defined by three or more indicators, two-indicator factors can be estimated in SEM when they are theoretically well defined and appropriately identified through parameter constraints [16]. In the present study, this specification was considered justifiable because both constructs were narrow, conceptually coherent, and measured through paired indicators that directly reflected their intended domains: working memory and inhibition for Executive Functions, and right- and left-leg balance for Postural Stability. To ensure model identification, standard scaling constraints were applied by fixing one factor loading per latent variable to 1.0 and estimating the remaining loading and error terms within the overall CFA/SEM framework. Accordingly, these two-indicator factors were treated as parsimonious latent representations of theoretically bounded constructs embedded within a broader identified model [16].

The decision to retain Executive Functions and Postural Stability as latent variables, rather than converting them to observed composite scores, was based on the conceptual advantages of latent modeling. Specifically, the SEM framework allowed the common variance shared by the paired indicators to be modeled explicitly, thereby reducing attenuation from measurement error and preserving consistency with the latent-variable treatment of the broader model.

The structural part of the model was specified to examine the relationships among executive functions, motor performance, physical activity, and anthropometric status in preschool children. During model development, several lifestyle and anthropometric variables, including physical activity, screen time, sleep duration, healthy eating score, body mass index, waist circumference, age, and sex, were considered based on their theoretical relevance and previously reported associations with cognitive and motor development in childhood. A covariance between the two motor latent variables was specified because of the expected overlap between different dimensions of motor performance.

The final SEM specification was determined using a theory-informed and parsimonious approach that prioritized variables most closely aligned with the study aims and conceptual framework. Physical activity and waist circumference were retained as the primary explanatory variables because they were central to the study hypotheses and demonstrated meaningful relationships with the motor and executive-function constructs. Age and sex were retained as a priori covariates because of their established developmental relevance. Screen time, sleep duration, healthy eating score, and body mass index were explored during model development but were not included in the final SEM specification because they were not central to the primary research questions and did not meaningfully improve model interpretation. Thus, the final model was intended as a theoretically grounded representation of the relationships most relevant to the study aims rather than as the result of a purely data-driven variable selection process.

Two residual covariances were specified in the final measurement model: one between right- and left-hand grip strength and another between catching and throwing. These covariances were not introduced as purely empirical corrections, but as theoretically justified allowances for shared task-specific and method-related variance. Right- and left-hand grip strength are bilateral measures of the same neuromuscular capacity obtained using the same instrument and procedure and therefore were expected to share variance beyond that explained by the broader Strength–Coordination factor. Similarly, catching and throwing are closely related object-control tasks that involve overlapping perceptual-motor demands and shared method characteristics. Including these residual covariances allowed the model to account for this specific overlap while preserving the broader latent structure of motor performance.

### 2.5. Statistical Analysis

All analyses were performed using Python (version 3.11.7) and its associated statistical libraries, including pandas, NumPy, scikit-learn, and semopy, for data management, statistical analyses, and structural equation modeling. Statistical significance was set at *p* < 0.05 (two-tailed).

Continuous variables were screened for out-of-range values and missing data prior to analysis. Missing data were low overall in the analytic variables, representing 0.35% of all data cells, with 6.04% of participants having at least one missing value. Missingness was concentrated primarily in age (5.49%), whereas the remaining variables showed either no missing values or only isolated missingness. Given this low proportion, missing values were imputed using the median of each variable to preserve the full sample for SEM estimation. Although full information maximum likelihood or multiple imputation are generally preferred in SEM, the low level of missingness in the present study suggests that any impact on parameter estimates was likely minimal. Sprint time was reverse-coded so that higher scores consistently reflected better motor performance across all indicators. Before latent-variable modeling, all continuous variables were standardized as z-scores to facilitate interpretation of standardized coefficients.

Descriptive statistics, including means and standard deviations, were calculated for all study variables. The latent structure of the measurement model was then evaluated by confirmatory factor analysis (CFA). Particular attention was given to the adequacy of the two-indicator factors representing Executive Functions and Postural Stability. Because such factors can be more sensitive to model misspecification than latent variables with a larger number of indicators, they were retained only within a theoretically constrained measurement model and interpreted cautiously [16]. Factor loadings were inspected to verify that all retained indicators loaded in the expected direction and were conceptually consistent with their corresponding latent constructs.

After the measurement model was established, a structural equation model (SEM) was estimated to examine the direct and indirect relationships among physical activity, waist circumference, motor performance, and executive functions, while adjusting for age and sex. Models were estimated using maximum likelihood. The sample size was considered adequate for SEM estimation given the moderate model complexity and favorable participant-to-parameter ratio.

During measurement-model development, several alternative specifications were evaluated. Tiptoe walking and hopping were initially considered as potential indicators of motor performance; however, these variables showed comparatively weak contributions to the latent structure and were associated with less satisfactory model fit. Therefore, they were excluded during model refinement to obtain a more parsimonious and theoretically coherent measurement model. The final specification retained only indicators that demonstrated adequate theoretical relevance and empirical performance within the SEM framework.

Model fit was evaluated using multiple complementary indices, including the chi-square statistic, the comparative fit index (CFI), the Tucker–Lewis index (TLI), and the root mean square error of approximation (RMSEA). Because global fit indices may vary in sensitivity according to model complexity, degrees of freedom, sample size, and model specification, model adequacy was interpreted from the overall pattern of fit indices rather than from any single cut-off value. In line with commonly used SEM guidelines, CFI values ≥ 0.90 were considered indicative of acceptable fit, RMSEA values ≤ 0.06 were interpreted as good approximate fit, and TLI values approaching 0.90 were considered acceptable in applied SEM settings, particularly when supported by other fit indices and theoretical coherence. Because the chi-square statistic is highly sensitive to sample size, it was interpreted cautiously and not used as the sole criterion of model adequacy.

Indirect effects were examined using nonparametric bootstrapping with 10,000 resamples. To complement the latent SEM findings, indirect-effect estimates were derived using product-of-coefficients procedures based on composite representations of the retained latent domains. Bias-corrected 95% confidence intervals were calculated, and indirect effects were considered statistically significant when the confidence interval did not include zero. Total, direct, and indirect effects were examined to distinguish between direct associations and indirect statistical associations involving the motor domain.

To improve transparency of SEM reporting, bivariate correlations, the covariance matrix used for model estimation, fit comparisons of alternative model specifications, and residual diagnostics for the final model were additionally summarized in the Supplementary Material.

## 3. Results

### 3.1. Descriptive Statistics

The descriptive characteristics of the sample are presented in Table 1. The final sample consisted of 364 children, with a mean age of 5.47 years (SD = 0.93), and 53.0% were boys. Mean values for physical activity, anthropometric indicators, executive function scores, and motor-performance measures are also shown in Table 1.

### 3.2. Measurement Model

A confirmatory factor analysis supported a three-factor latent structure comprising Executive Functions, Postural Stability, and Strength–Coordination. The final measurement and structural model, after inclusion of two theoretically justified residual covariances, showed acceptable fit to the data: χ^2^(68) = 139.46, *p* < 0.001; CFI = 0.915; TLI = 0.882; RMSEA = 0.054.

All retained indicators loaded in the expected direction on their corresponding latent factors. Working memory and inhibition loaded on Executive Functions; right- and left-leg balance loaded on Postural Stability; and standing long jump, grip strength, inverted sprint, catching, and throwing loaded on Strength–Coordination. Standardized factor loadings for the retained indicators of the final measurement model are presented in Table 2. These results supported the decision to treat balance-related performance and broader strength–coordination performance as separate motor domains, providing a conceptually clearer representation of the motor construct than a single undifferentiated motor factor.

The two-indicator latent factors for Executive Functions and Postural Stability were retained because both indicators loaded in the expected direction and were consistent with the theoretically bounded nature of these constructs.

As supplementary evidence supporting the internal consistency of the two-indicator latent constructs, the paired indicators showed moderate-to-strong positive correlations. Working memory and inhibition were strongly correlated (r = 0.698, *p* < 0.001), whereas right- and left-leg balance were moderately to strongly correlated (r = 0.547, *p* < 0.001) (Supplementary Table S1).

### 3.3. Structural Model

The structural model showed that physical activity positively predicted Postural Stability (β = 0.099, *p* = 0.024), whereas waist circumference negatively predicted Postural Stability (β = −0.096, *p* = 0.032). Age was positively associated with both motor latent variables, and sex was associated with both factors as well. In contrast, neither physical activity nor waist circumference significantly predicted Strength–Coordination.

**Figure 1:**
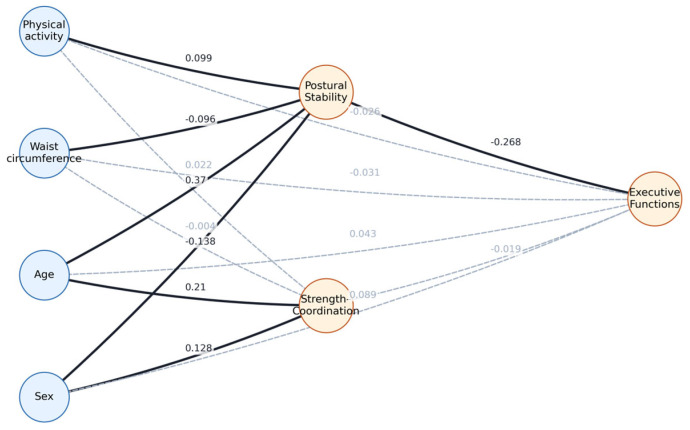
Final structural equation model. Values shown are standardized coefficients. Solid lines indicate statistically significant paths, and dashed lines indicate non-significant paths. CHEXI scores were coded so that higher values indicate greater executive difficulties; therefore, negative coefficients toward Executive Functions indicate better executive functioning.

Most importantly, Postural Stability was significantly associated with Executive Functions (β = −0.268, *p* = 0.016), whereas Strength–Coordination was not (β = −0.019, *p* = 0.912). Because higher CHEXI scores indicate greater executive difficulties, the negative coefficient for Postural Stability indicates that better balance performance was associated with better executive functioning. Direct paths from physical activity and waist circumference to Executive Functions were not statistically significant. The final structural model is illustrated in Figure 1, and the full set of standardized structural path coefficients is presented in Table 3.

The final model explained 20.6% of the variance in Postural Stability, 17.6% in Strength–Coordination, and 9.6% in Executive Functions.

### 3.4. Indirect Effects

Bootstrapped indirect-effect analyses indicated that the pathway from waist circumference to Executive Functions through Postural Stability was statistically significant (indirect β = 0.013, 95% CI [0.003, 0.035], *p* = 0.048). In contrast, the indirect effect of physical activity through Postural Stability was in the expected direction but only marginal (indirect β = −0.014, 95% CI [−0.037, 0.003], *p* = 0.051). No relevant indirect effects were observed through Strength–Coordination. Bootstrap estimates of total, direct, and indirect effects are presented in Table 4.

Bivariate correlations among the main observed variables are presented in Supplementary Table S2, and the covariance matrix used for model estimation is provided in Supplementary Table S3. In general, the balance indicators were associated with executive-function indicators and with physical activity and waist circumference in the expected directions, providing descriptive support for the SEM specification. In the final model, the explained variance was 20.6% for Postural Stability, 17.6% for Strength–Coordination, and 9.6% for Executive Functions. Comparison with alternative specifications showed that the final two-factor motor model provided better overall fit than both a one-factor motor model and a two-factor model without correlated residuals (Supplementary Table S4). Residual diagnostics for the final SEM are summarized in Supplementary Table S5.

As a sensitivity analysis, the final SEM was re-estimated using full information maximum likelihood (FIML) to evaluate the robustness of the findings to the handling of missing data. The key structural coefficients were highly consistent with those obtained using the original median-imputation approach. In particular, the associations between physical activity and Postural Stability, waist circumference and Postural Stability, and Postural Stability and Executive Functions remained similar in magnitude and statistical significance. Overall, the pattern of results and substantive conclusions was unchanged (Supplementary Table S6).

Taken together, the results suggest that the relationship between motor performance and executive functions in preschool children was more strongly related to the postural stability domain than to strength–coordination performance. In addition, postural stability appeared to represent the motor domain most closely associated with the links between waist circumference and potentially physical activity and executive functioning.

## 4. Discussion

The present study examined the relationships among executive functions, motor performance, physical activity, and waist circumference in preschool children using a structural equation modeling approach. Three main findings emerged. First, the association between motor performance and executive functions was domain-specific: executive functions were significantly associated with postural stability, whereas the latent factor representing strength–coordination was not. Second, physical activity and waist circumference were significantly associated with postural stability, but not with strength–coordination. Third, waist circumference showed a significant indirect association with executive functions through postural stability, whereas the corresponding indirect association for physical activity was only marginal. Taken together, these findings suggest that, in early childhood, the relationship between movement and executive functioning may depend less on overall motor performance and more on specific balance-related regulatory processes.

The main finding of this study was that postural stability, but not strength–coordination, was associated with executive functions. This result extends previous literature showing positive associations between motor competence and executive functions in children and adolescents [3,15], while adding specificity by suggesting that not all motor domains contribute equally in preschool children. Recent meta-analytic evidence indicates a small but reliable overall association between motor competence and executive functions across childhood [3,15], and longitudinal evidence further suggests that motor competence is associated with broader cognitive development over time [25]. However, many previous studies have relied on composite motor scores, making it difficult to determine which specific motor components may be most relevant. Our findings suggest that stability-related performance may be especially important during the preschool years.

Although the association between Postural Stability and Executive Functions was statistically significant, the proportion of variance explained in Executive Functions was relatively modest (R^2^ = 9.6%). Therefore, the present findings should not be interpreted as suggesting that motor performance is a major determinant of executive functioning in preschool children. Rather, they indicate that postural stability represents one of several factors that may be associated with everyday executive functioning during early childhood. Executive functions are complex, multidimensional processes that are likely influenced by a wide range of biological, environmental, educational, family, and behavioral factors that were not captured in the present model. Consequently, the practical importance of the findings lies less in the magnitude of the explained variance and more in the identification of a specific motor domain that appears to be more closely related to executive functioning than broader strength–coordination performance.

This interpretation is developmentally plausible. Postural stability is not merely a low-level motor output; it requires continuous integration of visual, vestibular, and proprioceptive information, anticipatory adjustments, error detection, and suppression of ineffective responses. In addition, postural stability may reflect aspects of neuromuscular control and dual-task processing, both of which have been linked to cognitive functioning and may contribute to the observed association with executive functioning. These characteristics overlap conceptually with executive control processes, especially inhibitory control and working memory updating [5,9]. In early childhood, when both motor and prefrontal systems are still undergoing rapid maturation, balance-related tasks may place particularly strong demands on self-regulatory processes. By contrast, the latent strength–coordination factor in the present study, although developmentally relevant, may reflect broader physical performance without the same degree of moment-to-moment cognitive regulation. This may explain why it was not significantly associated with executive functions in the final model.

It should also be noted that postural stability in the present study was represented primarily by static balance tasks. Although these tasks require continuous sensory integration and self-regulation, dynamic balance and movement-based postural control may place additional demands on executive processes because they involve ongoing adaptation, online motor adjustments, and regulation of behavior in changing contexts. Therefore, future research should examine whether dynamic forms of postural regulation show similar or even stronger associations with executive functioning during early childhood.

A second important finding was that physical activity positively predicted postural stability, whereas waist circumference negatively predicted postural stability. These results are consistent with prior work suggesting that active children tend to exhibit better motor performance and that greater adiposity may be associated with poorer movement quality and postural control [4,6,7,14]. However, in the present study these associations were specific to postural stability, as neither physical activity nor waist circumference significantly predicted strength–coordination. This pattern reinforces the idea that balance-related performance may represent a more sensitive correlate linking lifestyle and body composition with executive functioning in early childhood.

The finding that postural stability was negatively associated with CHEXI scores also requires careful interpretation. Because higher CHEXI scores indicate greater executive difficulties, the negative path coefficient observed in the model indicates that better postural stability was associated with better executive functioning, not worse. This distinction is important because the direction of association depends on the scoring of the executive-function measure. Thus, the results should be interpreted as showing that children with better balance performance tended to exhibit fewer everyday problems related to working memory and inhibition. It is also important to consider the nature of the executive-function measure used in this study. The CHEXI assesses parent-reported executive-function difficulties as expressed in everyday situations rather than objective executive performance measured through standardized neuropsychological tasks. Consequently, the observed associations should be interpreted as relationships between motor performance and caregivers’ perceptions of children’s executive-function difficulties in daily life. While ecological measures such as the CHEXI provide valuable information about real-world functioning, they may capture somewhat different aspects of executive functioning than laboratory-based performance measures. Future studies would benefit from combining questionnaire-based and performance-based assessments to provide a more comprehensive evaluation of executive functioning during early childhood.

With regard to indirect effects, the association between waist circumference and executive functions through postural stability was statistically significant, whereas the corresponding indirect association for physical activity was only marginal. This means that the evidence more clearly supports an indirect statistical association involving postural stability for central adiposity than for physical activity in this sample. Specifically, greater waist circumference was associated with poorer postural stability, which in turn was associated with more executive-function difficulties. In contrast, although higher physical activity was associated with better postural stability and the indirect effect was in the expected direction, the confidence interval for this association still included zero, indicating that the evidence was not sufficient to support a clearly significant indirect effect. Accordingly, the physical-activity association should be interpreted cautiously as suggestive rather than definitive.

It is important to note that these indirect effects represent statistical associations observed within a cross-sectional model and should not be interpreted as evidence of causal mediation. Although the observed pattern is consistent with the possibility that postural stability may be involved in the association between waist circumference and executive functioning, longitudinal and experimental studies are required to establish temporal ordering and determine whether these associations reflect causal developmental processes.

These findings partially align with prior research showing beneficial associations of physical activity and exercise with executive function in children [4,6,8,26]. At the same time, they suggest that in preschool children the relationship between physical activity and executive functions may be more closely related to balance-related regulatory processes than to broader motor-performance domains. This interpretation is also compatible with research indicating that the cognitive correlates of movement may depend not only on the amount of activity performed, but also on the coordinative, adaptive, and cognitively engaging demands imposed by the activity itself [5,8,26].

The present study has several strengths. It used a latent-variable approach that allowed executive functions and motor performance to be modeled at the construct level, reducing measurement error and permitting the simultaneous examination of direct and indirect associations. It also distinguished between two theoretically meaningful motor domains rather than relying on a single global motor score, which provided a more nuanced understanding of how motor performance relates to executive functioning in preschool children.

Several limitations should also be acknowledged. First, the cross-sectional design precludes causal inference, and therefore the directionality of associations should be interpreted cautiously. Second, executive functioning was assessed using the CHEXI, a parent-report questionnaire that captures executive-function difficulties as expressed in everyday contexts rather than objective executive performance measured through neuropsychological tasks. Although the CHEXI has demonstrated acceptable psychometric properties in preschool populations [14], parent-reported measures may reflect caregiver perceptions and contextual influences. Therefore, the findings should be interpreted as associations with parent-reported executive-function difficulties rather than direct measures of executive performance. In addition, executive-function difficulties were reported exclusively by parents. Future studies may benefit from incorporating multiple informants, including teachers, who observe children in structured educational settings and may provide complementary perspectives on everyday executive functioning. Third, physical activity and other lifestyle variables were parent-reported, which may introduce measurement error. An additional limitation concerns the handling of missing data. Although the overall proportion of missingness was low, missing values were addressed using median imputation rather than more robust SEM-oriented procedures such as full information maximum likelihood or multiple imputation. In addition, although the analyses adjusted for age and sex, other potentially relevant factors were not available in the present dataset. Variables such as socioeconomic status, parental education, participation in organized physical activities, characteristics of the home environment, and broader aspects of developmental maturity may influence both motor performance and executive functioning during early childhood [9,26]. Consequently, residual confounding cannot be ruled out, and the observed associations should be interpreted within the context of these unmeasured factors. Therefore, some caution is warranted, although the low level of missing data suggests that any resulting bias was likely small. Another limitation concerns the measurement structure of the model. Two latent constructs, Executive Functions and Postural Stability, were represented by only two indicators each. Although two-indicator latent variables can be identified and estimated when supported by strong theoretical rationale and appropriate parameter constraints [16], they are generally less robust than factors defined by three or more indicators. Accordingly, these constructs should be interpreted as relatively parsimonious latent representations, and future studies would benefit from including a broader set of indicators for both executive and postural-control domains. Nevertheless, the paired indicators defining each construct showed moderate-to-strong intercorrelations, providing supplementary evidence of their internal coherence. In addition, overall model fit, although acceptable, was not optimal, as reflected by a TLI value slightly below the conventional 0.90 threshold. Therefore, the SEM findings should be interpreted as theoretically grounded but still requiring replication in independent samples. Finally, participants were recruited using a convenience sampling strategy from a limited number of preschool centers within a specific geographic context. Therefore, the findings may not be fully generalizable to preschool populations from other regions, educational settings, or socioeconomic backgrounds. In addition, although age was included as a covariate in all structural models, the age range of 4–7 years encompasses important developmental changes in both motor and executive functions. Therefore, future studies should examine whether the observed associations differ across narrower developmental stages or are moderated by age.

Overall, the present findings indicate that the relationship between motor performance and executive functioning in preschool children is not uniform across motor domains. Instead, postural stability appears to be the motor component most consistently associated with executive functioning in this age group and the motor domain most closely related to waist circumference, and potentially physical activity, in relation to everyday executive functioning.

## 5. Conclusions

In preschool children, the association between motor performance and executive functions appeared to be more closely related to postural stability than to broader strength–coordination performance. Better postural stability was associated with better executive functioning, whereas strength–coordination showed no meaningful association with executive functions in the final model.

In addition, physical activity and waist circumference showed indirect associations with executive functions through postural stability. The indirect association between waist circumference and executive functions through postural stability was statistically significant, whereas the corresponding indirect association for physical activity was marginal and should therefore be interpreted cautiously.

These findings suggest that balance-related motor processes may be associated with executive functioning during early childhood. Although the observed associations were modest in magnitude, postural stability appeared to be more closely related to executive functioning than broader strength–coordination performance in preschool children. Future longitudinal and experimental studies are needed to determine whether these associations reflect developmental processes and to clarify the nature of the relationship between postural stability and executive functioning during early childhood.

## Figures and Tables

**Table 1:** Descriptive characteristics of the sample.

Variable	M	SD
Age (years)	5.47	0.93
Sex (boys), n (%)	193 (53.0%)	
BMI (kg/m^2^)	17.68	2.64
Waist circumference (cm)	58.54	7.59
Physical activity (h/week)	2.15	1.61
Screen time (h/day)	2.33	1.09
Sleep duration (h/day)	9.55	1.17
Healthy eating score	7.23	1.94
Working memory (CHEXI)	29.29	8.47
Inhibition (CHEXI)	30.91	7.5
Balance right leg (s)	16.55	10.06
Balance left leg (s)	16.95	10.16
Standing long jump (cm)	84.03	24.86
Grip strength right (kg)	7.87	3.68
Grip strength left (kg)	7.76	5.25
10 m sprint (s)	17.67	2.85
Catching (n)	7.65	2.45
Throwing (n)	4.83	2.62
Tiptoe walk (n)	13.92	3.15
Hopping (n)	4.82	0.97

**Table 2:** Standardized factor loadings for the final measurement model.

Factor	Indicator	Estimate	Std. Err	z-Value	*p*-Value
Executive Functions	Working memory (CHEXI)	1	-	-	-
Executive Functions	Inhibition (CHEXI)	0.952	0.205	4.655	<0.001
Postural Stability	Balance right leg (s)	1	-	-	-
Postural Stability	Balance left leg (s)	0.901	0.102	8.846	<0.001
Strength–Coordination	Standing long jump (cm)	1	-	-	-
Strength–Coordination	Grip strength right (kg)	1.202	0.188	6.408	<0.001
Strength–Coordination	Grip strength left (kg)	0.649	0.153	4.237	<0.001
Strength–Coordination	Inverted 10 m sprint (higher = better)	1.156	0.183	6.309	<0.001
Strength–Coordination	Catching (n)	1.238	0.194	6.382	<0.001
Strength–Coordination	Throwing (n)	0.807	0.165	4.886	<0.001

Standardized factor loadings are presented together with standard errors, z-values, and *p*-values.

**Table 3:** Standardized structural path coefficients for the final structural equation model.

Outcome	Predictor	Estimate	Std. Err	z-Value	*p*-Value
Postural Stability	Physical activity (h/week)	0.098	0.043	2.254	0.024
Postural Stability	Waist circumference (cm)	−0.095	0.044	−2.145	0.032
Postural Stability	Age (years)	0.370	0.047	7.826	<0.001
Postural Stability	Sex (boys = 1)	−0.137	0.063	−2.168	0.030
Strength–Coordination	Physical activity (h/week)	0.022	0.028	0.769	0.441
Strength–Coordination	Waist circumference (cm)	−0.004	0.028	−0.140	0.887
Strength–Coordination	Age (years)	0.210	0.037	5.640	<0.001
Strength–Coordination	Sex (boys = 1)	0.128	0.043	2.961	0.003
Executive Functions	Physical activity (h/week)	−0.025	0.049	−0.515	0.606
Executive Functions	Waist circumference (cm)	−0.031	0.050	−0.619	0.535
Executive Functions	Age (years)	0.042	0.061	0.693	0.488
Executive Functions	Sex (boys = 1)	0.089	0.078	1.139	0.254

Values are standardized coefficients. CHEXI scores were coded such that higher values indicate greater executive difficulties; therefore, negative coefficients for Executive Functions should be interpreted as reflecting better executive functioning.

**Table 4:** Total, direct, and indirect effects from bootstrap analyses.

Predictor	Total Effect	Direct Effect	Indirect Effect	95% CI	*p*-Boot
Physical activity (h/week)	−0.046	−0.030	−0.014	−0.037–0.003	0.051
Waist circumference (cm)	−0.010	−0.024	0.013	0.003–0.035	0.048

Indirect effects were estimated using 10,000 nonparametric bootstrap resamples. Confidence intervals represent 95% bootstrap confidence intervals. Indirect effects were considered statistically significant when the confidence interval did not include zero.

## Data Availability

The data presented in this study are available on request from the corresponding author. The data are not publicly available due to ethical standards.

## Supplementary Materials

The following supporting information can be downloaded at: https://www.mdpi.com/article/10.3390/children13070898/s1, Table S1: Correlations between indicators of the two-indicator latent constructs; Table S2: Correlations; Table S3: Covariance matrix; Table S4: Alternative model fit comparison; Table S5: Residual diagnostics for the final structural equation model; Table S6: Sensitivity analysis comparing the original SEM and the FIML re-estimated model.

## Abbreviations

The following abbreviations are used in this manuscript:

BMI	Body Mass Index
CFA	Confirmatory Factor Analysis
CHEXI	Childhood Executive Functioning Inventory
CFI	Comparative Fit Index
EF	Executive functions
ICC	Intraclass Correlation Coefficient
MABC-2	Movement Assessment Battery for Children, second edition
RMSEA	Root Mean Square Error of Approximation
SEM	Structural Equation Modeling
TLI	Tucker–Lewis Index
