# Supplementary material for: Postural Stability, Rather than Strength–Coordination, Is Associated with Executive Functions in Preschool Children: A Structural Equation Modeling Study
Source: Children (Basel). 2026 Jul 6;13(7):898. doi: 10.3390/children13070898 (PMC13407149; doi:10.3390/children13070898)
Supplement: Supplementary file 1 [file children-13-00898-s001.zip › children-4358302-supplementary.pdf]

Table S1. Correlations between indicators of the two-indicator latent constructs.

Construct	Indicator 1	Indicator 2	N	Pearson's r	95% CI	p-value
Executive Functions	Working memory	Inhibition	364	0.698	0.061 – 0.747	< 0.001
Postural Stability	Right-leg balance	Left-leg balance	364	0.547	0.471 – 0.616	< 0.001

Table S2. Correlations.

Variable	AGE	SEX	PA	WC	WM	IN	BR	BL	LJ	GR	GL	CAT	THR	10S
AGE	1	-0.066	0.045	0.198	-0.044	-0.100	0.359	0.330	0.313	0.235	0.191	0.195	0.231	0.176
SEX	-0.066	1	-0.081	-0.017	0.107	0.072	-0.129	-0.110	0.057	0.157	0.055	0.075	0.021	0.070
PA	0.045	-0.081	1	-0.014	-0.092	-0.020	0.133	0.100	0.069	0.004	-0.002	-0.030	0.095	0.045
WC	0.198	-0.017	-0.014	1	0.019	-0.066	-0.043	0.010	-0.089	0.198	0.077	-0.015	0.043	0.031
WM	-0.044	0.107	-0.092	0.019	1	0.698	-0.201	-0.070	0.013	0.037	-0.038	-0.104	-0.082	-0.055
IN	-0.100	0.072	-0.020	-0.066	0.698	1	-0.201	-0.093	0.036	-0.029	-0.023	-0.125	-0.087	-0.022
BR	0.359	-0.129	0.133	-0.043	-0.201	-0.201	1	0.547	0.203	0.115	0.101	0.230	0.207	0.140
BL	0.330	-0.110	0.100	0.010	-0.070	-0.093	0.547	1	0.216	0.128	0.140	0.317	0.198	0.177
LJ	0.313	0.057	0.069	-0.089	0.013	0.036	0.203	0.216	1	0.174	0.201	0.217	0.245	0.301
GR	0.235	0.157	0.004	0.198	0.037	-0.029	0.115	0.128	0.174	1	0.337	0.390	0.146	0.399
GL	0.191	0.055	-0.002	0.077	-0.038	-0.023	0.101	0.140	0.201	0.337	1	0.111	0.211	0.103
CAT	0.195	0.075	-0.030	-0.015	-0.104	-0.125	0.230	0.317	0.217	0.390	0.111	1	0.146	0.334
THR	0.231	0.021	0.095	0.043	-0.082	-0.087	0.207	0.198	0.245	0.146	0.211	0.146	1	0.137
10S	0.176	0.070	0.045	0.031	-0.055	-0.022	0.140	0.177	0.301	0.399	0.103	0.334	0.137	1

Abbreviations: PA, physical activity; WC, waist circumference; WM, working memory; IN, inhibition; BR, right-leg balance; BL, left-leg balance; LJ, standing long jump; GR, right-hand grip strength; GL, left-hand grip strength; CAT, catching; THR, throwing; 10S, inverted 10 m sprint (higher = better).

Table S3. Covariance matrix.

Variable	AGE	SEX	PA	WC	WM	IN	BR	BL	LJ	GR	GL	CAT	THR	10S
AGE	0.82	-0.042	0.065	1.357	-0.338	-0.679	3.276	3.040	7.047	0.782	0.909	0.432	0.549	0.453
SEX	-0.042	0.478	-0.09	-0.088	0.626	0.374	-0.899	-0.770	0.971	0.398	0.199	0.126	0.038	0.139
PA	0.065	-0.090	2.588	-0.170	-1.249	-0.240	2.154	1.641	2.745	0.022	-0.019	-0.119	0.400	0.206
WC	1.357	-0.088	-0.17	57.517	1.232	-3.776	-3.289	0.762	-16.708	5.509	3.056	-0.284	0.859	0.672
WM	-0.338	0.626	-1.249	1.232	71.761	44.385	-17.097	-6.048	2.743	1.160	-1.684	-2.159	-1.813	-1.333
IN	-0.679	0.374	-0.24	-3.776	44.385	56.314	-15.200	-7.120	6.740	-0.797	-0.913	-2.297	-1.710	-0.462
BR	3.276	-0.899	2.154	-3.289	-17.097	-15.200	101.290	55.962	50.854	4.242	5.328	5.668	5.453	4.012
BL	3.040	-0.770	1.641	0.762	-6.048	-7.120	55.962	103.179	54.577	4.766	7.467	7.884	5.278	5.131
LJ	7.047	0.971	2.745	-16.708	2.743	6.740	50.854	54.577	617.865	15.885	26.213	13.203	16.001	21.292
GR	0.782	0.398	0.022	5.509	1.160	-0.797	4.242	4.766	15.885	13.524	6.518	3.509	1.412	4.174
GL	0.909	0.199	-0.019	3.056	-1.684	-0.913	5.328	7.467	26.213	6.518	27.614	1.425	2.907	1.541
CAT	0.432	0.126	-0.119	-0.284	-2.159	-2.297	5.668	7.884	13.203	3.509	1.425	5.979	0.938	2.328
THR	0.549	0.038	0.400	0.859	-1.813	-1.710	5.453	5.278	16.001	1.412	2.907	0.938	6.880	1.023
10S	0.453	0.139	0.206	0.672	-1.333	-0.462	4.012	5.131	21.292	4.174	1.541	2.328	1.023	8.112

Abbreviations: PA, physical activity; WC, waist circumference; WM, working memory; IN, inhibition; BR, right-leg balance; BL, left-leg balance; LJ, standing long jump; GR, right-hand grip strength; GL, left-hand grip strength; CAT, catching; THR, throwing; 10S, inverted 10 m sprint (higher = better).

Table S4. Alternative model fit comparison.

Model	DoF	chi2	CFI	TLI	RMSEA	AIC	BIC
Final two-factor model	68	139.463	0.915	0.882	0.054	73.234	217.428
One-factor motor model	74	241.081	0.802	0.746	0.079	60.675	181.487
Two-factor model without residual covariances	70	153.001	0.902	0.866	0.057	69.159	205.560

CFI = Comparative Fit Index; TLI = Tucker-Lewis Index; RMSEA = Root Mean Square Error of Approximation; AIC = Akaike Information Criterion; BIC = Bayesian Information Criterion.

Table S5. Residual diagnostics for the final structural equation model.

Metric	Value
Mean absolute standardized residual	0.174
Median absolute standardized residual	0.153
Maximum absolute standardized residual	0.538
Residual pairs > 0.10	55
Residual pairs > 0.20	33

Table S6. Sensitivity analysis comparing the original SEM and the FIML re-estimated model.

Path	Original SEM	FIML SEM
Physical activity → Postural stability	0.126	0.126
Waist Circumference → Postural stability	-0.122	-0.122
Postural stability → Executive Functions	-0.244	-0.256
Strength-coordination → Executive Functions	-0.010	0.000

Values are standardized path coefficients. FIML = Full Information Maximum Likelihood; SEM = Structural Equation Modeling.
